# How beliefs about self-creation inflate value in the human brain

**DOI:** 10.3389/fnhum.2015.00473

**Published:** 2015-09-04

**Authors:** Raphael Koster, Tali Sharot, Rachel Yuan, Benedetto De Martino, Michael I. Norton, Raymond J. Dolan

**Affiliations:** ^1^Institute of Cognitive Neuroscience, University College LondonLondon, UK; ^2^Wellcome Trust Centre for Neuroimaging, Institute of Neurology, University College LondonLondon, UK; ^3^Affective Brain Lab, Department of Experimental Psychology Research, University College LondonLondon, UK; ^4^Department of Psychology, University of CambridgeCambridge, UK; ^5^Harvard Business School, Harvard UniversityBoston, MA, USA

**Keywords:** fMRI, amygdala, hippocampus, medial temporal lobe, caudate nucleus

## Abstract

Humans have a tendency to overvalue their own ideas and creations. Understanding how these errors in judgement emerge is important for explaining suboptimal decisions, as when individuals and groups choose self-created alternatives over superior or equal ones. We show that such overvaluation is a reconstructive process that emerges when participants believe they have created an item, regardless of whether this belief is true or false. This overvaluation is observed both when false beliefs of self-creation are elicited (Experiment 1) or implanted (Experiment 2). Using brain imaging data we highlight the brain processes mediating an interaction between value and belief of self-creation. Specifically, following the creation manipulation there is an increased functional connectivity during valuation between the right caudate nucleus, where we show BOLD activity correlated with subjective value, and the left amygdala, where we show BOLD activity is linked to subjective belief. Our study highlights psychological and neurobiological processes through which false beliefs alter human valuation and in doing so throw light on a common source of error in judgements of value.

## Introduction

Humans value their own creations (e.g., books, ideas, paintings, cupcakes) more highly than identical—and in some cases superior—products created by others (Franke et al., 2010; Norton et al., 2012) and believe other people will share their sentiment (Norton et al., 2012). The societal importance of these errors is that they can lead to suboptimal decision-making in sectors ranging from finance to health (Norton, 2009); for example, companies can overvalue the products they develop and physicians can favour objectively inferior medical procedures developed internally over superior ones developed elsewhere, the so-called “Not Invented Here” syndrome (Katz and Allen, 1982).

One possibility is that this overvaluation is a reconstructive process wherein a self-enhancing bias is implemented after the fact. In this case the mere belief^1^ that an item has been self-created should be sufficient to alter its value, irrespective of whether that belief is true or false. Indeed, on a wide range of skills and characteristics humans falsely believe they fare better than most other individuals and are better than they actually are, also known as the superiority illusion (Alicke et al., 1995; Dunning et al., 2003). Crucially, these self-enhancing valuations extend to entities associated with the self, including romantic partners (Murray, 1999), offspring (Weinstein, 1980), groups (Brewer, 1979), possessions (Nesselroade et al., 1999; Morewedge et al., 2009) and most relevant to the present study, self-created objects. The latter is known as the “Ikea effect” (Norton et al., 2012) or “I designed it myself” effect (Franke et al., 2010). While the existence of these biases has been extensively documented (Franke and Schreier, 2010; Franke et al., 2010; Mochon et al., 2012; Norton et al., 2012; Dohle et al., 2014; Kang and Lee, 2015), the cognitive and neural mechanisms by which overvaluation occurs is unknown.

Here, we examine whether this phenomenon is a result of a reconstructive process whereby a mere belief in self-creation of items—which we both measure and experimentally manipulate—alters people’s current estimate of the value of those items, regardless of whether the items are in fact self-created or not. We will use functional magnetic resonance imaging (fMRI) to identify a BOLD signal that tracks participants’ subjective value of items as well as subjective belief of creation. Using functional connectivity analysis we can then test whether and how these signals relate to each other. If overvaluation of self-created objects is related to an explicit belief of creation, neural signals indexing subjective belief (as identified within our task) may show enhanced synchronization with neural signals tracking value (as identified within our task) following creation, relative to before. The striatum has been shown in the past to process value (O’Doherty, 2004; Delgado, 2007), while subregions of the medial temporal lobe have been shown to index true and/or false belief (Eichenbaum et al., 2007; Edelson et al., 2011). Connectivity between those regions has frequently been associated with the interplay of decision making and memory (Pennartz et al., 2011; Wimmer and Shohamy, 2012). Here, we will first examine if those regions index value and explicit beliefs in our task, and then test for changes in functional connectivity between them.

**Figure 1:**
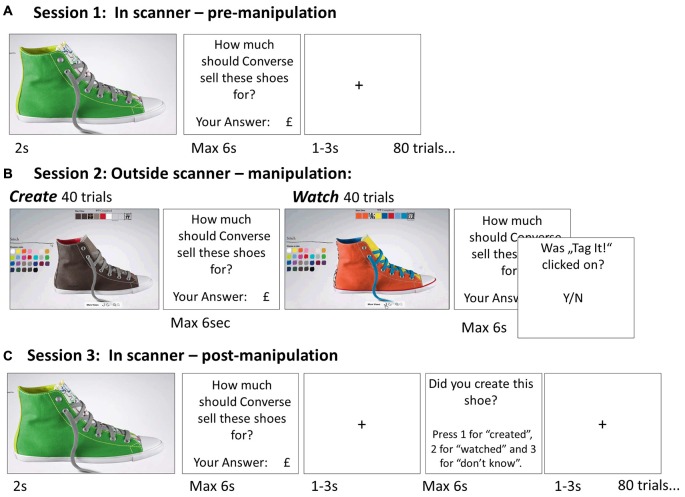
**Paradigm Experiment 1. (A)** Session 1 (pre-manipulation) was conducted in the fMRI scanner. On each of the 80 trials participants were presented with an image of a different Converse shoe and asked to key in their suggested retail price for that shoe using a button box. **(B)** Session 2 (manipulation session) was performed on a computer outside the scanner. There were 40 *create* trials interleaved with 40 *watch* trials. On each *create* trial participants were given specific instructions for creating one of the shoes presented in Session 1 (i.e., no customization allowed). The instructions were given as a series of colors and patterns that indicated which color/pattern was to be chosen for each part of the shoe (there were nine parts including laces, outer body, heel, etc.). The participants created the shoe on the Converse website (they were trained on creating shoes before they began) and clicked “save” when they were finished. On *watch* trials they viewed a video that portrayed the shoe being created on the website. To make sure they attended, at the end of each watch trial they indicated whether a “tag” button was clicked on the video. **(C)** Session 3 (post manipulation) was identical to Session 1, except that participants were also asked to indicate if they created the shoe in the previous session, watched it being created, or did not know.

**Figure 2:**
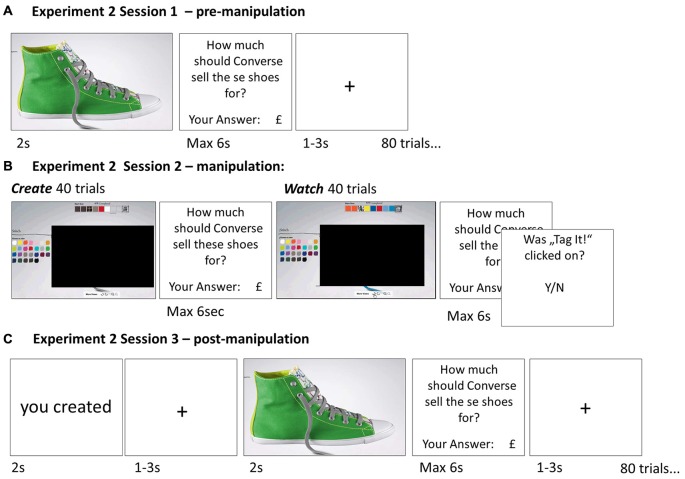
**Paradigm Experiment 2—Belief manipulation.** In order to manipulate subjects’ beliefs, rather than elicit them, we conducted a modified version of the task. Session 1 **(A)** of Experiment 2 was identical to Experiment 1. However, in Session 2 **(B)** the display of the actual shoe was occluded such that subject’s knowledge of which shoe was created and which was watched was more ambiguous and thus more easily manipulated. In Session 3 **(C)** before the shoe was displayed, subjects were instructed whether the shoe was created or watched in Session 2; this information was false half of the time.

To investigate how beliefs of self-creation are integrated into evaluation of items, we combined fMRI with a novel task in which participants evaluated 80 items before and after creating these pre-designed objects or watching the objects being created (Experiment 1, Figure 1). As the objects were pre-designed (equivalent to IKEA furniture) subjects could not design items according to their own preference. We included a large number of objects in our task so that subsequently participants would frequently hold false beliefs in regards to which items they created themselves and which they merely watched being created. This allowed us to dissociate the effect of creating an object from the mere belief in having created an object on changes in subjective value and brain activity. In other words, the paradigm enabled us to examine whether it is crucial that an individual create an item, or whether the mere belief in being instrumental in its creation is sufficient for overvaluation to take place, even when that belief is in fact false. In a second study (Experiment 2, Figure 2) we tested if beliefs of creation *cause* overvaluation by actively manipulating participants’ beliefs instead of eliciting them.

## Materials and Methods

### Participants

Twenty-three (Experiment 1) and 30 (Experiment 2) healthy right-handed participants who provided informed consent took part in the study. All had normal or corrected-to-normal vision and no history of mental illness. Participants were excluded that missed more than 10 value responses (one in Experiment 1 and four in Experiment 2). Additionally, in Experiment 1 two participants were excluded for having fewer than nine trials in at least one condition. This threshold was determined* a priori* (Sharot et al., 2004). The exclusion of the participants did not affect the pattern of the central behavioral finding (effect of belief: *F*_1,22_ = 5.89, *p* = 0.024; effect of reality: *F*_1,22_ = 0.59, *p* = 0.81; interaction: *F*_1,22_ = 3.17, *p* = 0.09). One participant was eliminated due to a technical error that resulted in loss of partial fMRI data leaving nineteen participants (nine female; 20–37 years, mean age 23.3 years) in Experiment 1 and twenty-six in Experiment 2 (13 female; 18–32 years, mean age 23.4 years). The study was approved by the Institute of Neurology (University College London) Research Ethics Committee. Participants provided informed consent and received reimbursement for their participation.

### Stimuli

In each study stimuli consisted of 80 pictures of Converse shoes, varying in colors and pattern but not model, brand or material. The stimuli were presented in randomised order on a gray background using Cogent 2000^2^ running in MATLAB.

### Procedure

#### Experiment 1

The study consisted of three consecutive sessions (Figure 1). In session 1 (pre-manipulation stage, ~20 min) and session 3 (post-manipulation stage, ~30 min) participants were asked to evaluate objects (shoes) while in an fMRI scanner. Session 2 (manipulation stage, ~80 min) was conducted outside the scanner, in which subjects created objects or watched objects being created. Each session began with instructions and example trials. Furthermore, subjects were supervised for at least the first few test trials to ensure compliance with the instructions.

### Session 1 and 3 (Pre-Manipulation and Post-Manipulation Sessions)

Before each session participants completed a number of training trials. Each item (shoe) was presented for 2 s. Participants were asked to advise the manufacturer at which price the shoe should be sold to customer “Matt Brown” (as in Norton et al., 2012) the task was to estimate the item’s worth to “an average Joe”. This pricing procedure measures how an individual estimate *others* will value an item. In other words, we examine if participants believe even other people should be willing to pay more for an item created by the participant. Participants had up to 6 s to answer on a scale from £11 to £88 using four button boxes in each hand (only eight buttons were available, thus numbers 0 or 9 could not be used). A fixation cross was then displayed for 1–3 s (jittered). In Session 3, participants were also asked whether they previously created or watched the shoe being created, or whether they don’t know. Finally, a second fixation cross was displayed for 1–3 s (jittered).

### Session 2 (Manipulation-Session)

Session 2 was conducted using a computer outside the scanner. There were 40 *create* trials interleaved with 40 *watch* trials. The items presented were those viewed previously in Session 1 (allocation to condition was counterbalanced).

#### Watch Trials

On *watch* trials participants viewed pre-recorded videos of a shoe being created on the Converse website. Thus, what the participant viewed on screen was identical to what he/she viewed on the computer screen while making a shoe themselves. Participants were then asked to evaluate the shoe (as in Session 1 and 3) and report whether or not the video featured the removal of a “tag” on the shoe (this feature of the shoe design is independent of the color). This question ensured that participants attended to the videos (average accuracy 91%).

#### Create Trials

On *create* trials participants created a shoe on the Converse website according to specific instructions resulting in a specific shoe. The instructions were presented as sequence of colors/patterns that conveyed information about which color/pattern each shoe part should be. In other words, participants could not create shoes according to their own preferences. Once the shoe was completed the participant clicked “save”. Participants were led to believe that the saved stimuli would be used in Session 3. In fact, for simplicity, identical stimuli saved previously by the experimenter were used. Participants were then asked to value the shoe (as in Session 1 and 3). The actual price of the shoe displayed on the website was occluded.

#### Experiment 2

Experiment 2 was identical to Experiment 1 except for the following: (1) All parts of the study were conducted outside the scanner; (2) The shoe was occluded during session 2 for both create and watch trials; and (3) During session 3, participants were informed whether they *created* or *watched* the shoe. The information was false half the time. Specifically, the words “you created” or “you watched” were displayed on screen for 2 s, then a fixation cross (jittered for 1–3 s) was displayed followed by the shoe image and the rating question.

### Analysis of Behavioral Data

In both experiments, for each participant within each session value ratings were *z* transformed (hereafter value) in order to account for non-specific effects across sessions such as participants becoming fatigued (see Sharot et al., 2009, 2010). The transformed value represents the relative value of that stimulus compared to all other stimuli for that participant in that session. Note that the same results are found for untransformed data. The *change* in value from Session 1 to Session 3 was then calculated for each participant in each condition and entered into a repeated measures ANOVA: 2 reality (watch/create) × 2 belief (watch/create, by participants’ expression of belief in Experiment 1 and instructed belief in Experiment 2). This analysis was repeated with the initial values from session 1 in order to examine dependence of initial value and later belief. In Experiment 1, trials on which the participant responded “don’t know” in response to whether they created or watched the shoe, and trials in which they did not enter a value in the allotted 6 s, were removed from the analysis (six trials on average). To investigate whether the RT of the belief question was associated with the value change, the correlation between measures was calculated within each participant. The average correlation coefficient was tested against zero with a one-sample *t*-test. The reaction times (RTs) to respond to the belief question were analyzed in the same 2 × 2 ANOVA described above.

### MRI Scanning

The study was conducted at the Wellcome Trust Centre for Neuroimaging at University College London using a 3T Siemens Allegra scanner. Functional scans used a gradient echo sequence optimised for orbitofrontal cortex and amygdala coverage (Weiskopf et al., 2007). Time of repetition (TR) = 2.52 s, time of echo (TE) = 30 ms, flip angle (FA) = 90°, matrix = 64 × 64, field of view (FOV) = 192 × 192 mm^2^, slice thickness = 2 mm. Forty two axial slices (–30° tilt) were sampled for whole brain coverage, in-plane resolution = 3 × 3 mm. An MPRAGE sequence was used to acquire structural images after session 3 which comprised 1-mm-thick axial slices parallel to the anterior commissure/posterior commissure plane. Imaging data were analyzed with SPM5 (Wellcome Trust Centre for Neuroimaging). Images were realigned with the first volume (after discarding six dummy volumes) and unwarped, normalized to the the Montreal Neurological Institute reference template, resampled to 2 × 2 × 2 mm^3^ voxels, and spatially smoothed (8 mm full width at half-maximum).

### Analysis of fMRI Data

For each participant a time series was created indicating the temporal position of: (1) Display of stimuli (shoes); (2) Motor responses; (3) Onset of the rating question; and (4) Fixation. These were modeled as time periods of 2, 0, 0, and the specific duration of each fixation period (average 2 s, respectively. In addition, a parametric modulator containing the *z*-transformed value rating was entered modulating the time period of shoe presentation. Time periods were convolved with the canonical hemodynamic response function. Motion correction regressors were entered as covariates of no interest.

The general linear model (GLM) of the third session was identical to session 1 above, but in addition it contained the onset of the belief question with three parametric modulators; (1) Distinguishing accurate from inaccurate belief; (2) Distinguishing participants’ belief of whether they created or watched the shoe being created; and (3) Distinguishing shoes that were in reality created from watched.

In order to identify regions that were activated at the time of the belief question activity during the time of belief question was contrasted against fixation (*p* < 0.05, FWE cluster level corrected after voxel-wise thresholding at *p* < 0.001, see Sharot et al., 2004, 2007b, for a similar procedure). Four regions emerged: left amygdala, left hippocampus, left and right occipital lobe, and right insula. To allow precise localisation of activation in the amygdala and hippocampus we constrained emerging voxels using anatomical masks via the WFU PickAtlas toolbox (Lancaster et al., 2000; Maldjian et al., 2003). We identified regions where BOLD signal correlated with item value using a parametric modulation analysis (value modulating the time period of shoe presentation) over both sessions (*p* < 0.05, FWE cluster level corrected after voxelwise thresholding at *p* < 0.001). Here, the caudate nucleus was of specific interest as it has previously been shown to be involved in value change due to action related to the self (Sharot et al., 2009, 2010) enabling on *a priori* grounds to use small volume correction for anatomically defined right and left caudate nucleus.

We implemented a second GLM to allow a 2 reality (watch/create) × 2 belief (watch/create) ANOVA. This was identical to the first GLM except that the item presentation and belief question were divided into five different conditions: Items that were; (1) Created and believed to be created (created-created); (2) Created and believed to have been watched (created-watched); (3) Watched and believed to be created (watched- created); (4) Watched and believed to be watched (watched- watched); and (5) Items for which the participant indicated they “don’t know”. Each presentation condition contained a parametric modulator containing the *z*-transformed rating. Additionally, in Session 3 another parametric modulator was included containing the *z*-transformed rating change.

For each participant the average beta estimates over all voxels during the shoe representation, in each of the four critical conditions, in each session, was calculated for the regions of interest (ROIs) identified in the first step of the analysis (those regions significantly more active during belief expression vs. fixation, and regions tracking value). Then the change in average betas between Session 1 and 3 was calculated and entered into a 2 reality (watch/create) × 2 belief (watch/create) ANOVA (using the change in betas as a dependent variable is equivalent to adding an additional “session” factor to the ANOVA with two levels). Average betas over all voxels in each of the ROIs were also calculated for the four critical conditions during the belief question presentation in Session 3. These were entered into a 2 reality (watch/create) × 2 belief (watch/create) ANOVA. Note that the identification of ROIs is completely independent from follow up ANOVAs. A psycho-physiological interaction (PPI) analysis was conducted to investigate the connectivity between the ROIs during the two sessions.

### Functional Connectivity Analysis

In the first model, a PPI analysis was conducted to examine functional coupling between regions showing significant effects in the first analysis (i.e., the “seeds”; amygdala, hippocampus, insula) and those tracking value (i.e., the “targets”; caudate and inferior frontal gyrus, IFG) before and after the manipulation session at the time of shoe presentation. Three PPIs were implemented (i.e., one for each seed) where the regressors included: (1) The activation time course of the volume of interest (i.e., physiological variable); (2) A regressor representing the psychological variable of interest (the time of item presentation); and (3) A regressor representing the cross product of the previous two (the psychophysiological interaction term, PPI). The first two regressors were added as covariates to the model whilst the last regressor was of interest. For each participant, we extracted the parameter estimates of the PPI regressor for each of the target ROIs for the pre and post-manipulation sessions. Then we conducted paired *t*-tests to investigate if the functional connectivity has changed between the pre and post-manipulation sessions (Bonferroni corrected for six comparisons). In order to show that a change was not due to a general increase in connectivity over time, we conducted the same analysis on the whole time course of the scans (as in Sharot et al., 2010).

## Results

### Subjective Belief, Objective Reality and Value

**Figure 3:**
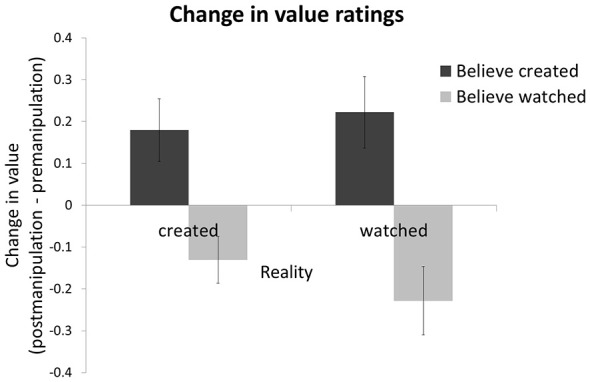
**Subjective belief, not objective reality, is related to value.** Change in the value participants assigned to items (price post-manipulation minus price pre-manipulation) was greater for shoes participants believed they had created than shoes they believed they had watched being created. Whether participants in fact created or watched the shoe being created had no effect on value change. A positive score represents an increase in subjective value after the manipulation session relative to before. Error bars show within-subject standard error of the mean (Cousineau, 2005).

Participants valued items more after the manipulation stage relative to before if they believed they created the item themselves, and this was the case whether this belief was veridical or not. Specifically, entering value *change* (rating of value after manipulation minus rating before) into a 2 (subjective belief: created/watch) by 2 (objective reality: create/watch) ANOVA revealed a main effect of subjective belief (*F*_1,18_ = 6.522, *p* < 0.05, with no effect of reality or interaction of reality and belief). The effect remains also after *z*-transforming the data within each session in order to remove non-specific session effects like fatigue and reduced attention over time (*F*_1,18_ = 10.07, *p* < 0.01; Figure 3). The increase in value after the manipulation session was significant for items the participants actually created and correctly believed they created (t_18_ = 2.3, *p* < 0.05), but equally so for items participants had watched being created but then falsely believed they had created (*t*_18_ = 2.6, *p* < 0.05). There was no main effect of objective reality (*F*_1,18_ = 0.18, *p* = 0.68), indicating that whether participants created an item or watched it being created was not related to value change. We observed no interaction (*F*_1,18_ = 1.42, *p* = 0.25) such that belief accuracy (i.e., whether participants’ belief matched reality) was not related to value change. Thus, creation* per se* was not associated with participants’ evaluation, whereas subjective belief in having created an item enhanced value.

Participants were instructed to press “create” and “watch” only if they had a sense that they created the item or watched it being created, otherwise they should press the “don’t know” button, which they did 7% of the trials. The task was designed to induce a large number of false responses so that belief could be dissociated from reality. Indeed, out of the trials in which participants indicated their beliefs, they were inaccurate on 47.5% of the trials. To assure subjects’ were not randomly pressing buttons we compared the rate of the three types of responses to chance. All were significantly different from chance (“created”: *t*_18_ = 5.09, *p* < 0.001; “watched”: *t*_18_ = 6.05, *p* < 0.001; “don”t know’: *t*_18_ = 10.12, *p* < 0.001, chance level at 33%). Together, these results suggest that subjects were complying with the instruction to indicate a belief. We emphasize that these beliefs do not need to indicate a true memory.

### Did Participants’ Belief in having Created an Item Enhance the Item’s Value or did Participants Simply Believe They Created Objects They Valued Highly?

Examining subjects’ initial rating of items in Rating Session 1 did not reveal an effect of belief, reality, nor an interaction (all *p* > 0.17). In other words, while the value of items they believed to have created was enhanced following our experimental manipulation, *these were not the items they initially rated highly*. Could random increases in value over the two sessions trigger a belief that these items were created? To test this possibility, we conducted a second experiment on a new set of participants, in which we actively manipulated participants’ beliefs rather than assessed them.

To manipulate participants’ beliefs, we altered the task slightly so that veridical recollection of actual creation of an item was even less likely than in Experiment 1. Specifically, we occluded the shoe during the manipulation session on both creation trials and watch trials (see ““Materials and Methods” Section for details). Participants could still create the shoes as before, by clicking the correct button on the color panels that described the shoes design, and watch the creation process by observing the button being clicked. However, without observing the shoes during this stage it was more difficult for participants to form veridical beliefs of which shoe was created and which was watched. In the final session, participants were told before viewing each shoe whether they created the shoe (“you created”) or whether they watched it being created (“you watched”). Crucially, this information was false half of the time, allowing us to experimentally dissociate belief from actuality and test the causal impact of belief on value. The results of Experiment 2 revealed a main effect of instructed belief on value change (*F*_1,25_ = 4.23, *p* = 0.05), participants valued shoes more after they were told they had created them relative to before. There was no main effect of reality (i.e., whether the shoe was actually created or watched did not affect value, *F*_1,25_ = 0.01, *p* = 0.97) and no interaction (i.e., whether the instruction matched reality did not affect value, *F*_1,25_ = 2.27, *p* = 0.14).

After the final part of Experiment 2, participants completed a short questionnaire consisting of five questions probing whether they had any suspicion that the information displayed to them was random. The questions were “funnelled” to get increasingly more sensitive and suggestive of the fact that the displayed information was random. We used the final, most sensitive question (“In fact the information was wrong half of the time, did you notice that?”) to split the sample into subjects with no suspicions at all and subjects with any suspicion at all. The debriefing questionnaire showed that 10 of 26 subjects had some suspicion that the given information (“you watched”/”you created”) was random. This number is similar to previous studies using deception (Edelson et al., 2011, 2015). Examining data of subjects who reported no suspicion strengthened our results: effect of belief: *F*_1,15_ = 8.19, *p* = 0.01, with no effect of reality: *F*_1,15_ = 0.36, *p* = 0.56 and no interaction: *F*_1,15_ = 2.25, *p* = 0.15. This suggests that participants in the task did not just associate the shoes they liked with themselves, but that believing they had a hand in creation enhanced perceived value.

Together these results suggest that overvaluation of self-created items is a reconstructive process which is contingent on a belief in having been the author of the creation.

### fMRI Results

The behavioral results revealed a relationship between explicit belief and valuation, leading us to use fMRI data to characterise brain mechanisms mediating this link. To do so we first identified BOLD signals associated with subjective belief and veridical representations of reality and then identified BOLD signal that tracked value. After these regions were identified, we asked the critical question of how these types of signals relate to each other after the creation manipulation.

### Representation of Belief and Reality

**Figure 4:**
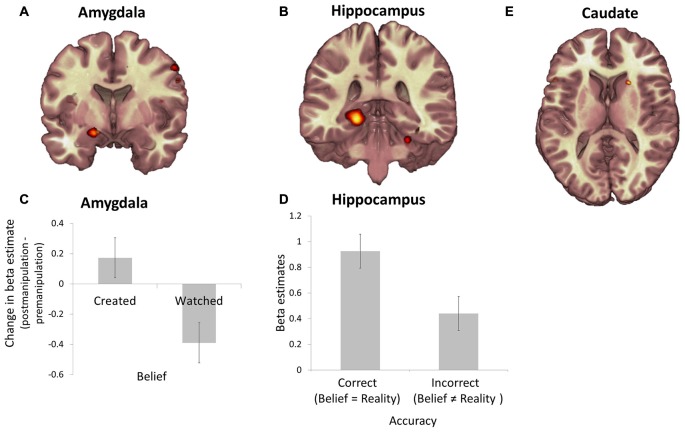
**Representation of subjective belief, veridical belief and value in the amygdala, hippocampus and caudate.** Regions in which the BOLD response was significantly enhanced during belief expression relative to fixation included the **(A)** left amygdala and **(B)** left hippocampus (all *p* < 0.05, cluster level corrected). **(C)** During item presentation *change* in BOLD response during session 3 (post-manipulation) relative to session 1 (pre-manipulation) averaged across the voxels in the left amygdala was greater during presentations of shoes participants believed they created relative to shoes they believed they watched. **(D)** In contrast, BOLD response during belief expression across the voxels in the left hippocampus was greater during trials in which participants gave an accurate response (veridical belief of whether they created the shoe) relative to trials in which they gave an incorrect belief judgment. **(E)** BOLD response in the right caudate nucleus during shoe presentation over both sessions correlates with the value participants assigned to the shoes (*p* < 0.05 FWE, small volume corrected). Error bars show within subject standard error of the mean (Cousineau, 2005).

First, to identify brain regions engaged at the time of belief expression we used an approach employed by us previously (Sharot et al., 2004, 2007b); conducting a broad search for any voxels where activity was significantly enhanced at the time participants indicated their belief (over all trials) relative to all fixation (*p* < 0.05, FWE cluster corrected). Significant effects were observed in four regions; the left amydgala (MNI coordinates of peak value, 16 4 −16, *k* = 155; Figure 4A), right insula (34 −18 18, *k* = 226), left hippocampus (−22 −32 −4, *k* = 618; Figure 4B) and bilateral occipital lobe (−10 −94 −12, *k* = 7453).

We then set out to identify which of the three regions was signaling belief, which reality and which accurate recollection (i.e., where belief matched reality). Note, that this approach allows definition of the function of each ROI within our specific task and data thus we do not need to rely on reverse inference (we do not examine occipital lobe further as activity here is likely related to visual activation, see Wandell et al., 2007).

As our main behavioral finding showed that belief in creation was associated with an enhancement in value assigned to an object following the manipulation, we first examined for parallel changes in BOLD activity by estimating the *change* in average beta (after manipulation—before manipulation) for each subject across all voxels, for each condition and region, during object presentation. These were entered into a 2 (belief: created/watch) by 2 (reality: create/watch) ANOVA. The analysis revealed a main effect of belief in left amygdala (*F*_1,18_ = 4.49, *p* < 0.05) and an effect of reality in insula (*F*_1,18_ = 8.06, *p* < 0.05). Specifically, *change* in activity across sessions in the amygdala was significantly greater for items believed to have been created relative to items believed to have been watched (Figure 4C). In the insula activity changes across sessions was significantly reduced for items participants watched being created relative to those they had themselves created.

There were no significant changes in left hippocampal activity across sessions. What then was the function of the hippocampus in this task? We tested if BOLD activity in the hippocampus was related to belief accuracy during the time of belief expression. Indeed, this was evident in a significant interaction between belief and reality (*F*_1,18_ = 4.73, *p* < 0.05). As displayed in Figure 4, we observed greater activity in the hippocampus for trials where belief matched reality (i.e., trials that were created and believed to be created and trials that were watched and believed to have been watched) than for trials where belief was false (Figure 4D). This suggest that there was a trace of accurate (possibly implicit) memory, a notion that was supported by analysis of RTs.

Interestingly, participants’ implicit responses in Experiment 1 reflected a trace of veridical reality. Specifically, entering the time it took (reaction time/RT) participants to indicate their belief of having created or watched the item being created into a 2 (belief: created/watched) by 2 (reality: create/watched) ANOVA revealed a main effect of reality (*F*_1,18_ = 4.86, *p* < 0.05). Participants were slower to respond when presented with items they created compared to those they watched, regardless of explicit belief. These longer RTs may reflect a greater amount of information retrieval for created items—such as past processing of creation instructions. Importantly, this implicit representation of reality was not related to value change (there was no correlation between value change and RT; across participants average *r* = −0.02, t_18_ = 0.48, *p* = 0.64) and RT were dissociated from participants’ explicit response as evident by the fact that there was no main effect of belief on RT and no interaction between belief and reality on RT. This result is consistent with previous results demonstrating dissociations between implicit behavior and explicit responses, with the former providing more accurate indication of past occurrences (Hannula and Ranganath, 2009).

This first part of the analysis was successful in identifying the regions were activity was related to belief and reality. We thus turned to the crucial question of how these representations relate to changes in value signal.

### Relationship between Value, Belief, and Reality

To index regions tracking value we conducted a parametric modulation analysis across all trials in both sessions to identify voxels where activity correlated with participants’ rating of value. This revealed activity during item presentation in right caudate nucleus (a-priori ROI, FWE small volume corrected *p* < 0.05; 20, 18, 10; *k* = 11; Figure 4E) and left IFG (−52, 14, 2; FWE cluster corrected *p* < 0.05, *k* = 305) correlating with participants’ ratings of value. No other regions in the brain showed a significant effect; we emphasize that neither amygdala, insula nor hippocampus activity tracked value.

**Figure 5:**
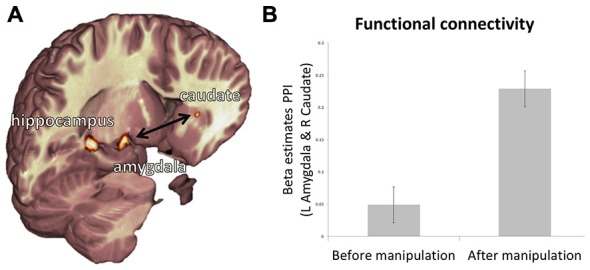
**Relationship between representations of value, belief, and reality. (A)** Depiction of functional ROIs of the left hippocampus, left amygdala and right caudate. **(B)** PPI beta estimates reveal that functional connectivity from pre-manipulation session to post-manipulation session is increased between the left amygdala and right caudate. Error bars show within subject standard error of the mean (Cousineau, 2005).

Next, we examine how activity in the regions tracking value (i.e., caudate and IFG) relates to activity associated with subjective belief, reality and veridical belief (amygdala, insula, and hippocampus). We assessed changes in functional connectivity between regions mediating these processes during item representation after the manipulation stage relative to before. Psychophysical interaction analysis (Figure 5A) revealed a significant increase in functional connectivity between the amygdala and caudate after the manipulation phase relative to before (Figure 5B; t_18_ = 3.22, *p* < 0.05, Bonferroni corrected for six multiple comparisons between the two ROIs indexing value, and the three ROIs related to belief expression). This increase was specific to the time of object presentation, and not to the entire time-course of the BOLD response (t_18_ = 0.18, *p* = 0.89). There were no other significant effects. These results mirror our behavioral findings, which indicate that subjective belief, not reality* per se*, is associated with value following creation.

## Discussion

Our results demonstrate that when people create items they come to overvalue their creations. These errors in judgement could have societal impact, as individuals and groups may come to overvalue inferior projects and ideas in sectors ranging from business to health. Our results reveal a psychological and system level mechanism by which such valuation interacts with belief of self-creations. The data shows that overvaluation of self-creations is not contingent on customization of items (see also Norton et al., 2012), as our experimental design does not allow participants to customize items according to their individual preferences. Nor is it contingent on becoming familiar with the process by which an item is created, as our experimental design allows participants to observe the creation process in both “watch” and “create” conditions. In fact, our findings show that overvaluation is not even contingent on actually having created the item. Rather, such overvaluation appears to be a reconstructive process that can be manipulated, resulting in participants quickly re-evaluating items once they come to believe they have created them, whether that belief is true or false.

Participants did not simply believe they created objects they value more as evidenced by: (1) the initial value of objects participants subsequently believe to have created was not higher than those they subsequently believe they had watched being created; and (2) instructing participants on which objects they had created and which they had watched being created. In sum, people’s belief about their self-creation actions in the past, rather than objective reality, was a significant contributor to shaping value. This was true irrespective of whether participants’ beliefs were elicited or manipulated, and suggests that actual effort need not be invested in an object for its subjective value to increase. This, however, is not to say that investing effort may not enhance value even further. The dependence on retrospective and explicit belief, independent of reality, is a novel insight into the nature of overvaluation. Using brain imaging, we identify BOLD signals that relate to subjective value and subjective belief in this task. This approach allows definition of the function of each ROI within our specific task and thereby obviates the need to rely on reverse inference. We reveal increased functional connectivity between these signals following the creation manipulation. Specifically, following creation, participants’ explicit beliefs about the prior occurrence of the creation event were associated with alteration of left amygdala activity. The association between amygdala activity and subjective belief is consistent with previous reports suggesting that activity in the amygdala signals subjective aspects of recollection, such as vividness or confidence, that can be dissociated from reality (Sharot et al., 2004; Dolcos et al., 2005; Edelson et al., 2011). Importantly, amygdala activity not only varied according to subjective belief, but was increasingly correlated with activity in the right caudate, which tracked the value of items. This correlation was seen only after the manipulation session, when belief of self-creation interacted with evaluation, and this linkage was not evident before. The involvement of the caudate in tracking value in this context is of interest as it aligns with evidence that this region is involved in the representation of a goal based value (Wunderlich et al., 2012), consistent with the inferential nature of the processing required in our task.

Amygdala activity is often dissociated from the pattern of activity observed in adjacent hippocampus (Sharot et al., 2004; Dolcos et al., 2005; Kensinger, 2009) and surrounding cortices (Sharot et al., 2004, 2007a). Such dissociations are observed when emotional and/or motivational factors alter subjective aspects of recollection independently of the accuracy of the judgment (Sharot et al., 2004, 2007a; Dolcos et al., 2005; Edelson et al., 2011). Here, while amygdala activity reflected participants’ beliefs, heightened activity in the left hippocampus was only observed when participants provided accurate belief judgements. This signal did not show enhanced functional connectivity with a caudate value signal, consistent with the fact that accuracy of beliefs did not drive value. In future work, investigation of the relative involvement of the amygdala’s subregions could be of interest, due to their varied projections and involvement in decision-making (Boureau and Dayan, 2011). Furthermore, using this design with different stimuli will enable to generalize the findings to other types of creation. Our investigation shows that subjective belief can be closely associated with preference, even in situations where those beliefs are false. In this context, the amygdala and dorsal sectors of the striatum appear to play a key role in mediating an interaction between preference and belief. Our neural results suggest overvaluation due to creation is a process that integrates subjective belief into value judgements as the judgement is being made. These results provide a possible mechanism by which false beliefs can alter human evaluation, potentially leading to errors in judgment.

## Conflict of Interest Statement

The authors declare that the research was conducted in the absence of any commercial or financial relationships that could be construed as a potential conflict of interest.

## Footnotes

^1^We use the term “belief” as per its Oxford dictionary definition; “an acceptance that a statement is true or that something exists”.

^2^www.vislab.ucl.ac.uk/cogent.php
